# Phage Display Libraries for Antibody Therapeutic Discovery and Development

**DOI:** 10.3390/antib8030044

**Published:** 2019-08-23

**Authors:** Juan C. Almagro, Martha Pedraza-Escalona, Hugo Iván Arrieta, Sonia Mayra Pérez-Tapia

**Affiliations:** 1 GlobalBio, Inc., 320, Cambridge, MA 02138, USA; 2 UDIBI, ENCB, Instituto Politécnico Nacional, Prolongación de Carpio y Plan de Ayala S/N, Colonia Casco de Santo Tomas, Delegación Miguel Hidalgo, Ciudad de Mexico 11340, Mexico; 3 CONACyT-UDIBI, ENCB, Instituto Politécnico Nacional, Prolongación de Carpio y Plan de Ayala S/N, Colonia Casco de Santo Tomas, Delegación Miguel Hidalgo, Ciudad de Mexico 11340, Mexico

**Keywords:** naive repertoires, synthetic repertoires, antibody library design, developability, hit rate, affinity

## Abstract

Phage display technology has played a key role in the remarkable progress of discovering and optimizing antibodies for diverse applications, particularly antibody-based drugs. This technology was initially developed by George Smith in the mid-1980s and applied by John McCafferty and Gregory Winter to antibody engineering at the beginning of 1990s. Here, we compare nine phage display antibody libraries published in the last decade, which represent the state of the art in the discovery and development of therapeutic antibodies using phage display. We first discuss the quality of the libraries and the diverse types of antibody repertoires used as substrates to build the libraries, i.e., naïve, synthetic, and semisynthetic. Second, we review the performance of the libraries in terms of the number of positive clones per panning, hit rate, affinity, and developability of the selected antibodies. Finally, we highlight current opportunities and challenges pertaining to phage display platforms and related display technologies.

## 1. Introduction

The phage display methodology has revolutionized the protein engineering field, with a major impact on antibody engineering. The first breakthrough in the remarkable development of phage display methodology, which became one of the most successful technology platforms for engineering antibody-based drugs, was published in 1985 by George Smith [1]. Smith showed that foreign DNA fragments could be inserted into the gene encoding the filamentous phage coat protein III (pIII), expressed as peptide-phage fusions and enriched more than 1000-fold over wildtype phage by affinity to a specific antibody directed against the peptide. Three years later, Parmley and Smith [2] described how peptide-phage fusions in a proportion as low as one peptide-phage fusion in a million wildtype virions could be enriched by using biotinylated antibodies specific for the peptides, a method coined as biopanning. Subsequently, three groups [3,4,5] showed that libraries of millions of random short peptides yielded specific peptides that could tightly bind to diverse antibodies used as selectors, thus laying the foundations for identifying specific peptides for any given antibody with no prior knowledge of its specificity.

**Figure 1:**
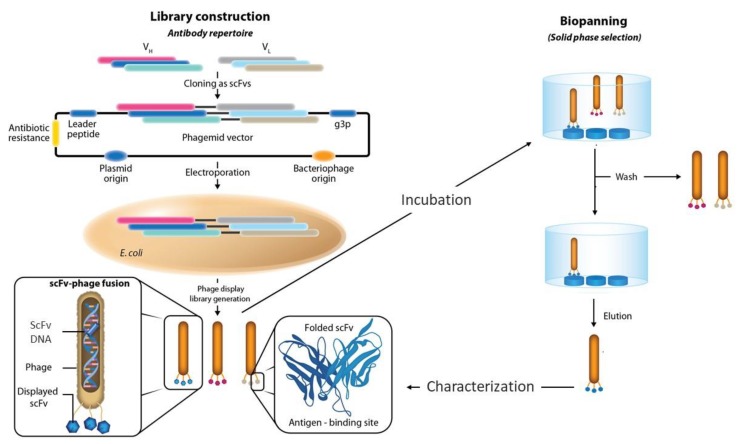
Phage display methodology. The method consists of building a library (left panel) of peptide or protein variants—or in the case of antibodies, an antibody gene repertoire—and affinity selecting (right panel) specific antibody-phage fusions via affinity with the target of interest. An antibody library is commonly built in a phagemid vector as fusions to one of the phage coat proteins. The repertoire of antibody variants, for instance cloned in the scFv format, is used to transform *E. coli* via electroporation and is expressed together with the other viral components. The population of specific scFvs-phage particles is enriched via binding to the target of interest. Non-specific or not-well-folded scFvs are removed via washing steps. The specific scFv-phage fusions are then eluted using diverse elution methods. The eluted phage bearing specific scFv genes can be used to infect *E. coli* and thus amplify the population of specific antibodies via additional rounds of selections. Alternatively, the outcome of the selection is characterized in the screening phase.

By the beginning of the 1990s, John McCafferty and Gregory Winter [6] extended the applications of the phage display methodology to antibody engineering. The genes coding for the variable (V) regions of the well-known murine anti-hen egg-white lysozyme (HEL) antibody D1.3 [7] were inserted into pIII and displayed on the phage surface as fusion proteins. D1.3-phage fusions were then isolated via HEL-affinity chromatography, similar to the specific peptides described in earlier works [3,4,5]. Further studies showed that as long as the pIII-fusions were not toxic to *Escherichia coli*, the phage display methodology was agnostic toward what type of protein [8] or antibody [9,10] genes were inserted into the phage coat proteins, displayed on the phage surface and selected with specific antibodies or ligands (Figure 1). This disruptive advantage of phage display with respect to the hybridoma technology developed almost two decades before [11] had a major impact on the development of therapeutic antibodies. While hybridoma technology was limited to obtaining monoclonal rodent antibodies, phage display enabled the isolation of human antibodies, hence minimizing the immune response associated with nonhuman antibodies when used in human therapy [12].

Because the phage display selection process is performed in vitro, this new technology platform also enabled the isolation of therapeutic antibodies in a variety of settings. For instance, necitumumab, a human antibody to treat cancer, was obtained by competition with the chimeric antibody cetixumab using phage display [13]. Another example is Adalimumab, the first fully human antibody to reach the market and the bestselling drug worldwide. Adalimumab was discovered and optimized using phage display via guided selection with a murine antibody [14]. In fact, up until 2017, six fully human therapeutic antibodies [15] discovered and/or engineered via phage display were approved by the Food and Drug Administration (FDA) and/or the European Medicines Agency (EMA), and hundreds have made it to clinical trials [16].

Initially, the main goal of phage display antibody discovery campaigns was to select for specific and high affinity antibodies [17]. In the last decade however, it has been learned that in addition to the specificity and affinity, other properties account for the success of a therapeutic antibody in clinical trials. Such properties, collectively called developability [18,19], include (but are not limited to) cross-reactivity against the human target and orthologs from relevant tox species and animal models, solubility, expression yield in manufacturing cells, and thermal and long-term stability. Since these properties are encoded in the amino acid sequence of the antibody, there has been a strong demand to generate phage display antibody libraries that not only enable the selection of highly specific and high affinity antibodies, but also those that are more developable.

As a result, numerous phage display antibody libraries have been built after the seminal work by John McCafferty and Gregory Winter in 1990 [6]. These libraries, their construction process, validation with diverse targets, and applications of the selected antibodies to diagnostic and/or therapeutic settings have been extensively reviewed in the literature [20,21,22,23,24,25]. Here, given the vast amount of information pertaining to phage display, compounded with space limitations, we review nine phage display scFv and Fab libraries published in the last decade (Table 1). These nine libraries are the latest generation of phage display antibody libraries and represent the state-of-the-art platforms for human therapeutic antibody discovery. We first discuss the functionality of the libraries in terms of size, quality, and diversity of the antibody repertoires. Second, we review and compare the outcome of the selection processes, i.e., the number of positive clones, hit rate, and affinity of the selected antibodies. Third, we discuss the performance of the libraries in terms of the developability of the selected antibodies. Finally, we highlight current opportunities and challenges relating to phage display platforms and other display technologies.

## 2. Size of the Phage Display Antibody Libraries

The correlation between the size of an antibody library and the likelihood of selecting for the desired antibodies is somewhat intuitive, i.e., the larger the antibody library, the higher the chances of isolating more diverse and higher affinity antibodies, and hence, the higher the possibility of selecting the desired molecule. Alan Perelson [34,35] formalized this concept as *p* = e^−N*p*^, where P is the probability that a given antibody does *not* recognize an epitope of a random shape, N is the size of the antibody library, and *p* is the probability that said antibody contacts said epitope with certain affinity. If *p* = 5 µM (5 × 10^−6^ M), which is a weak dissociation constant, but measurable and differentiable from non-specific binding, a library of N = 10^6^ antibodies would yield a *p* = 6.8 × 10^−3^ (≈2.7^−(1,000,000 × 0.000005)^). This means that the universe of virtually all possible random epitopes would be recognized by an antibody with affinity around 5 µM in a library of one million antibody variants. To reach the same (low) *p* value but with a dissociation constant of 5 nM (5 × 10^−9^ M), N should be 10^9^ antibody variants. Hence, the larger the library, the higher the chances that an antibody will specifically bind a random epitope with higher affinity.

The maximum value that N can reach, or the universe of all possible unique antibody variants, is virtually infinite. The antigen-binding site is made of six complementarity-determining regions (CDRs)—three in V_L_: LCDR1, LCDR2, and LCDR3, and three in V_H_: HCDR1, HCDR2, and HCDR3, which alternate with relatively conserved regions called framework regions (FRs) in the V domains. Considering the Kabat’s definition [36] of CDRs and the most frequent CDR lengths in humans [37]: LCDR1 = 11, LCDR2 = 6, LCDR3 = 8, HCDR1 = 4, HCDR2 = 19, and HCDR3 = 12, the CDRs are on average ten amino acids in length. If each position of each of the six CDRs that conform the antigen-binding site were diversified with the 20 natural amino acids, the corresponding theoretical repertoire would contain 20^(6 × 10)^ = 20^60^ or 1.2 × 10^78^ unique antibody variants. Obviously, only a very small fraction of this universe of 10^78^ unique antibody variants can be sampled in a phage display antibody library. On the other hand, practical factors limit the maximum size of a phage display antibody library to 10^10^–10^11^ antibody variants. These factors are: (1) the transformation efficiency of *E. coli*, which is between 10^10^ and 10^11^ colony transforming units (cfu) per µg of DNA (e.g., see the electrocompetent cells in the ThermoFisher catalogue); and (2) the volume needed to expand the library in cases where multiple electroporations are performed to increase the number of cfus after the ligation process.

All of the libraries listed in Table 1 reached on average 10^11^ cfu, with sizes ranging from 1.5 × 10^10^ cfu (Human Antibody gene Libraries, HAL9/10) to 3.6 × 10^11^ cfu (XscFv2). Therefore, all the libraries reviewed here have (or are close to) the maximum possible N. Consistent with the Perelson’s principle, these libraries produced single nM or sub-nM antibodies when panned with a diverse array of targets, as discussed later in the review.

## 3. Effective Size of the Phage Display Antibody Libraries

Ideally, the size of a library should be equal to its effective size, meaning that all the 10^11^ antibody variants comprising the library are displayed as functional antibody molecules on the phage surface. Nonetheless, the quality of the gene synthesis, either using RT-PCR (Reverse transcription polymerase chain reaction) for naïve libraries [9] or using chemical means for synthetic and semisynthetic libraries [29,38], affects the effective size of the libraries. Nucleotide sequences with stop codons leading to truncated sequences do not produce functional antibody fragments fused to a virion particle. Insertions or deletions of one or two nucleotides change the reading frame of the gene sequence generating truncated sequences, with stretches of amino acids that may impair folding or clones with solvent-exposed hydrophobic amino acids resulting in aggregation. Moreover, some in-frame antibody genes have low expression due to suboptimal codon usage in *E. coli* and/or inefficient translocation in the cell compartments [39]. These variants are displayed in low proportion with respect to other variants or are not displayed at all on the phage surface, which further erodes the effective size of the libraries. Furthermore, library synthesis methods based on mixtures of oligonucleotides or degenerated NNK codons might result in a reduction of diversity at the amino acid level due to the redundancy of the genetic code and/or encoded unwanted amino acids at targeted positions for diversification.

Table 2 summarizes the percentage of open reading frames (ORFs) and percentage of antibody expression or display of the libraries listed in Table 1. It should be noted that some authors do not report the percentage of ORFs and/or display, whereas, others measured these parameters in diverse ways. For instance, Xoma [26] calculated the percentage of ORFs based on the number of complete sequences between the start of signal sequences through to the end of both the V_L_ and V_H_ for XFab1 and from the start of the signal sequence through to the end of the V_L_ for XscFv2. The percentage expression (or display) was calculated based on the number of clones with an OD (optical density) over three-fold the background in an ELISA (Enzyme-Linked ImmunoSorbent Assay) for periplasmic expression of Fab or scFv. Kim et al. [28] reported as functional ORFs those sequences with no stop codons; they did not report the expression nor display before the selections. The authors of pIX V3.0 [29] estimated the percentage of ORFs as sequences without stop codons and functionality as Fab display in an ELISA. For HuCAL (Human Combinatorial Antibody Library) PLATINUM [30] and Ylanthia [31], MorphoSys analyzed sequence correctness by comparing the actual sequences with the design. Weber et al. (PHILODiamond, Phage Display Library Cloning [32]) studied the frequency of antibody-expressing clones using PCR and dot-blot analysis of the expressed Fabs. We (ALTHEA Gold Libraries™ [33]) reported ORFs as in-frame and non-stop codon sequences and display as Protein A binding in an ELISA.

Despite the diverse ways of measuring the functionality of the libraries, some meaningful trends can be highlighted. Except for pIX V3.0, which can be considered an outlier, the percentage of ORFs ranged from 66% in lambda XscFv2 to 93% in PHILODiamond. The display varies from 58% (also in XscFv2 lambda) to 90% (PHILODiamond). As expected, the display is slightly lower than the percentage of ORFs. Considering the percentage of display as the effective size of the libraries, the libraries of Table 1 have an effective size of 60–90%. The effective size is thus one order of magnitude below the absolute size of the libraries, i.e., 10^10^ antibody variants.

## 4. Types of Antibody Repertoires

While the library size can be defined by one number (N) and the effective size can be assessed by the percentage of antibody display, the diversity of a library has a somewhat elusive definition. Sometimes the size of an antibody repertoire is used as a proxy to characterize its diversity. However, it assumes that the antibody repertoire is a random set of antigen-binding site shapes and amino acid sidechains. Furthermore, not all antibody repertoires are created equal.

For instance, if one were to generate a library of antibody variants by diversifying all the 11 positions of the LCDR1 with the 20 amino acids, the resultant repertoire would be 20^11^ or 2 × 10^14^ antibody variants. This would appear to be a diverse repertoire based on its size and it would thus presumably lead to a large number of functional antibodies. Nevertheless, most of the amino acids in LCDR1 are located in the periphery of the antigen-binding site and have a marginal role in defining the binding properties of antibodies, especially for small and mid-sized targets [40]. Therefore, such a library would very likely be of limited use. In contrast, the same library size, e.g., 11 positions mutated to the 20 amino acids, but in the HCDR3, which is located at the center of the antigen-binding site and plays a critical role in recognizing diverse targets, would likely yield antibodies with reasonable affinity [41]. Hence, diversity is not only the number of in-frame and/or non-toxic antibody variants, but it is also the number of functional molecules capable of recognizing as many diverse targets as possible.

The first functional antibody repertoires used as a substrate to build phage display antibody libraries were obtained from immunized animals [42] or humans [43]. This type of library, known as immune, has been of limited application as general-purpose platforms for human therapeutic antibody discovery. Those obtained from immunized animals are single-use libraries as the libraries are biased toward the recognition of the antigen utilized as an immunogen. More importantly, the antibodies obtained from immune libraries, being nonhuman proteins, require further engineering (humanization), which increases the cost of the antibody drug development process. In the case of immune human antibody libraries, due to ethical reasons, they have mostly been used for isolation of antibodies against infectious diseases [44]. Nevertheless, it is worth noting that relatively recent studies of the immune repertoire of Camelid V regions have shown identical CDR conformations to those found in the human V germline gene repertoire [45]. This similarity has led to the development of a discovery platform by ArgenX based on the immunization of camelids. ArgenX has discovered several promising therapeutic antibodies that are difficult to obtain by other means [46], with some of them in advanced stages of clinical trials (https://www.argenx.com/en-GB/content/argenx-in-short/2/).

The first human universal or general-use library was published in the early 1990s [9]. This library was generated with the repertoire of human genes encoding the circulating antibodies. Libraries generated with this source of antibody genes have been called naïve as they are not biased toward any particular target. Although successful, naïve libraries included antibody genes toxic to *E. coli*, which as discussed above, compromised the effective size of the library. Synthetic antibody libraries followed to partially mitigate this limitation [38]. In this alternative approach, the libraries were carefully designed by making assumptions on the number of scaffolds, positions to diversify, type of amino acids to include in the design, and the proportion of each amino acid per position to diversify. These assumptions did not always hold true, particularly at the HCDR3. To avoid making assumptions on the structure of the CDRs, a combination of synthetic scaffolds with natural CDRs have been used [47,48], leading to the construction of the called semisynthetic libraries.

Table 1 includes four naïve and four synthetic libraries, as well as one semisynthetic library. Two of the naïve libraries were developed by Xoma [26]; one as Fab (XFab1) and the another as scFv (XscFv2). A third naïve library was published by Kugler et al. [27] at the Technische Universität Braunschweig, Institut für Biochemie, Biotechnologie und Bioinformatik (TU-IB). It combines kappa (HAL10) and lambda (HAL9) light chains displayed as scFvs. The fourth and most recently published naïve library [28] was developed at Kangwon National University (KNU). It only contains kappa-type light chains, and thus we called it KNU-Fab. The synthetic libraries listed in Table 1 include two generations of libraries from MorphoSys: HuCAL PLATINUM [30] and Ylanthia [31]. Both are optimized versions of the initial HuCAL and HuCAL GOLD [38,49] libraries. Further, Table 1 lists one synthetic library from Janssen Biotherapeutics, namely pIX V3.0 [29]. This was the first Fab antibody library displayed as pIX instead of pIII fusions. The fourth synthetic library is PHILODiamond [32]. This is a minimalist library built on only three scaffolds, one V_H_ and two alternative V_L_s, one kappa and another lambda. PHILODiamond is the latest version of a series of antibody libraries of increasing size and complexity generated by Dario Neri’s laboratory over the last 20 years, e.g., ETH2 (Eidgenössische Technische Hochschule 2) [50], ETH2Gold [51], and PHILO-1 and PHILO-2 [52]. The semisynthetic library was called ALTHEA (from the Greek *“to heal”*) Gold Libraries [33]. It was published by our group in collaboration with Antibody Design Labs (ADL) and The Tri-Institutional Therapeutics Discovery Institute (TDI). ALTHEA Gold Libraries™ combined one V_H_ scaffold with two Vκ scaffolds. The diversity of the HCDR3 came from a large pool of 200 human donors. In the following sections, we describe in detail each of the naïve, synthetic, and semisynthetic libraries listed in Table 1.

### 4.1. Naïve Libraries

Naïve libraries do not make assumptions about the diversity of the antibody repertoire. The rationale is that the human antibody repertoire evolved to recognize any target with a reasonable specificity and affinity. Therefore, the goal when building naïve libraries is to mirror the diversity of the human antibody repertoire while avoiding biases and redundancy due to the immunological history of a few individuals and/or rare polymorphic antibody genes present in the repertoire of a given ethnic group.

Xoma used 30 ethnically diverse healthy donors and a variety of tissues to amplify the V_H_ and Vκ and V_λ_ chains using RT-PCR. The tissue samples included 20 peripheral blood mononuclear cell (PBMC) samples, eight bone marrow samples, one spleen sample, and one lymph node sample. The amplification strategy encompassed all the Immunoglobulin (Ig) classes: IgM, IgG, IgA, IgE and IgD. The libraries were displayed in Fab or scFv formats to assess potential differences in the selection of antibodies depending on the display format. Both libraries yielded a similar number of unique antibodies with similar affinity (discussed in more detail below), indicating that the display format did not significantly impact the outcome of the selections.

HAL9/10 also used blood samples from diverse ethnic groups, including Caucasian, African, Indian, and Chinese donors. The libraries, one kappa (HAL10) and the other lambda (HAL9), contained the same V_H_ repertoire obtained from 98 donors but differed in their light chain repertoires. HAL9 included all lambda subfamilies from the 98 donors, whereas HAL10 was generated with 54 donors and contained all kappa families except the IGKV7 pseudogene. The amplification strategy included a reverse primer for V regions derived from IgMs, thus favoring the amplification of naïve antibody genes, i.e., close to the germline configuration and hence with few or no somatic mutations in V_H_.

The KNU-Fab library was generated with a larger pool of donors than the Xoma and HAL9/10 libraries. It included 803 PBMC donors, two lymph node donors, two spleen donors, and two bone marrow donors, totaling 809 samples. Thirty-three PBMC samples were obtained from healthy Korean human donors and 770 samples were obtained from commercial samples, probably from donors of diverse ethnic backgrounds. The antibody genes were amplified using forward and reverse primers hybridizing in the V regions, and thus agnostic as to the Ig class, isotype, or whether the antibodies were close to the germline gene configuration or had a substantial number of somatic mutations.

**Figure 2:**
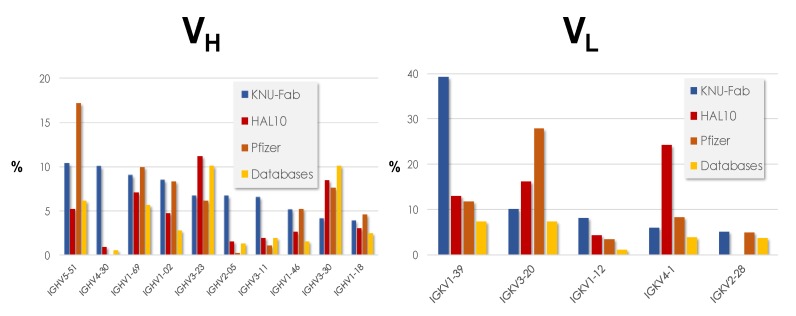
Use frequency of the most prevalent genes reported by KNU-Fab and HAL10. Glanville’s [53] study of the gene usage of Pfizer’s naïve library and antibody sequences collected in the antibody sequence databases is also shown.

The gene usage prior to the selections of the libraries was reported for KNU-Fab and HAL9/10 libraries, whereas for Xoma’s libraries, only the gene family usage was described. A total of 7373 unique V_H_ sequences and 41,804 unique Vκ sequences from the KNU-Fab library were studied to assess its diversity prior to selections with any target. Likewise, for HAL9/10, a total of 827 full length scFv sequences were analyzed from HAL9 and 466 sequences from HAL10. Figure 2 shows the frequency of the ten most prevalent IGHV (left) genes and the five most used IGKV (right) genes reported by KNU-Fab and HAL10. As a reference, we added Glanville’s [53] study of the gene usage of Pfizer’s naïve library and antibody sequence deposited in the databases.

The ten most prevalent IGHV genes are only 20% of ≈50 potentially functional IGHV genes in the human genome [54,55]. These ten genes covered 70% of KNU-Fab studied sequences and close to half (47%) of all the HAL9/10 studied sequences. The frequency of these IGHV genes follow a similar trend in all the samples, except the genes IGHV4-30, IGHV2-05, and IGHV3-11, which are overrepresented in KNU-Fab but have a low frequency in the other samples. Interestingly, genes from the IGHV4 family have been found to be selected negatively in other libraries [56] as they have been reported to be toxic to the B-cells [57]. In V_L_, five IGKV genes out of ≈35 [58] (14%) potentially functional IGKV genes in the human genome explained 68% of KNU-Fab sequences and 58% of HAL10 sequences. IGKV1-39 was overrepresented in KNU-Fab, comprising nearly 40% of all the sequences, whereas the IGKV4-1 gene was overrepresented in HAL10 with a frequency of close to 25%. Interestingly, the IGVK2-28 was expressed in all the samples except HAL10. Taken together, the highly skewed nature of the IGV gene usage in the naïve libraries indicate that only a few antibody genes are enough to cover the needed diversity to recognize diverse antigens in a phage display antibody library.

Regarding the HCDR3 diversity, all the four naïve libraries showed the typical Gaussian distribution of HCDR3 lengths observed in humans [59]. Both of Xoma’s libraries have a similar HCDR3 length distribution, with the most frequent lengths between 5 and 25 and an average length of 15 amino acids, in accordance with the Immunogenetic Database (IMGT^®^) definition [60], and 13 amino acids based on Kabat’s definition [36]. The HCDR3 of the HAL9 and HAL10 ranged from 5 to 35 amino acids, with low frequencies of long lengths (25–35 amino acids) and a median value of 14 amino acids (also IMGT’s definition) or 12 using Kabat’s definition. The KNU-Fab HCDR3 lengths ranged from 4 to 19 amino acids, with the most frequent HCDR3 loop lengths being 11 or 12 amino acids (Kabat’s definition).

In summary, to build the naïve libraries diverse pools of human donors were used. In some cases, the authors cast as wide a net as possible to amplify all the human genes, regardless the isotype or number of somatic mutations, for instance, in the Xoma and KNU-Fab libraries. In other cases, such as HAL9/10, the amplification strategy was constrained to sequences in the germline gene configuration. In all of the libraries, only a few antibody genes represented the bulk of the studied sequences, consistent with the patterns observed in the repertoire of human antibodies [53]. The HCDR3 length distribution was also similar in all the libraries and mirrored the Gaussian distribution typical of the human antibodies.

### 4.2. Synthetic Libraries

Synthetic repertoires are intended to maximize the functionality of the antibody libraries by using well expressed and developable scaffolds, targeting positions for diversification that do not disrupt folding of the V regions and selecting types and frequency of amino acids that facilitate selection of diverse binders to any given target. The first synthetic libraries, HuCAL and HuCAL GOLD [38,49], were developed by MorphoSys in the late 1990s. In common with its predecessor HuCAL GOLD, HuCAL PLATINUM (Table 1) was built with seven V_H_ and seven V_L_ master scaffolds, which when combined, yielded 49 antibody sub-libraries. These scaffolds were designed with consensus sequences representing the IGV genes families of the human repertoire. The sequences of the scaffolds were optimized for high expression in *E. coli* and display on the phage. The six CDRs were randomized using trinucleotide mutagenesis (TRIM) technology [61]. This synthesis technology is based on trimers encoding the 20 amino acids instead of mixes of oligonucleotides. In this way, the quality of the synthetic genes increased significantly since TRIM realized precise combinations of amino acids at targeted positions for diversifications while avoiding stop codons and unwanted amino acids that can disrupt the antibody folding.

One of the main differences between GOLD and PLATINUM are that the latter includes newly designed HCDR3 sequences. The new strategy was based on a systematic analysis of the amino acid usage per position of HCDR3 sequences in different loop lengths. Different amino acid frequencies were then used to design the synthetic HCDR3 fragments based on the specific length dependent amino acid use frequencies, instead of a relative uniform amino acid distribution for all HCDR3 of diverse length. In addition, PLATINUM used loop lengths from 4 up to 23 amino acids, covering over 95% of the naturally occurring HCDR3 lengths. Further, potential N-glycosylation sites generated in HuCAL and GOLD by the NXT/S pattern (where N is asparagine, X is any amino acid, and S/T is serine/threonine), were removed from PLATINUM. As a result of these changes, a side-by-side comparison of HuCAL GOLD and PLATINUM [30] indicated that the latter generated approximately 4-fold more unique and higher affinity antibodies.

Ylanthia [31] was yet another step in the MorphoSys path to enhance the performance of its antibody discovery platforms. Rather than consensus V_H_ and V_L_ master scaffolds, Ylanthia used selected V_H_:V_L_ combinations in the germline gene configuration. The selection process that led to the final design included the human germline genes most prevalent in the human antibody repertoire and those covering the canonical structure repertoire seen in the human antibodies. The canonical structures were discovered by Cyrus Chothia and Arthur Lesk [62] in the late 1980s. These authors found that, although the CDRs vary in sequence, five out of the six CDRs (LCDR1, LCDR2, LCDR3, HCDR1, and HCDR2) had a limited set of main-chain conformations or canonical structures. The canonical structure model has been updated in the last two decades [63,64] and helped to develop 3D modeling strategies [65]. From a structure–function perspective, the canonical structure model suggested that structural constraints are at work in antigen recognition [66]. Recently, the application of clustering algorithms [67] on 300 non-redundant antibody structures has further stratified the canonical structure combinations by identifying 28 combinations of CDR lengths with canonical structures, whereas previous analysis [63] covered only 20.

To build Ylanthia [31], this first in silico selection filter based on antibody gene usage and canonical structures generated 400 random V_H_:V_L_ combinations. This set of scaffolds were then experimentally tested in several developability assays. The assays assessed the expression level, thermal and serum stability, as well as aggregation propensity in Fab and IgG1 formats. Additionally, the initial V_H_:V_L_ combinations were tested for relative levels of Fab CysDisplay (a platform used by MorphoSys based on the expression of antibody-fragment linked to phage particles by a disulfide bond). After the experimental developability screening, 36 V_H_:V_L_ combinations, including 12 V_H_, 12 Vκ, and eight V_λ_ scaffolds, were used to build the libraries (Table 3).

In addition to this improvement with respect to the HuCAL series, Ylanthia’s CDRs were diversified based on a systematic analysis of a large set of rearranged human antibody sequences and potential developability liabilities. Moreover, a new synthesis technology known as Slonomics [68] was used in the generation of the HCDR3 sequences. Slonomics is a fully automated protein synthesis platform developed by Sloning Biotechnology and acquired by MorphoSys. This platform is based on sets of double-stranded DNA triplets coding for the twenty amino acids. The platform enables the highly controlled synthesis of diverse combinatorial gene libraries with high fidelity, i.e., closely matching the expected frequencies of designed amino acids with those observed in the libraries. As a TRIM technology, this new synthesis method avoided stop codons and unwanted mixes of amino acids in designed positions.

**Figure 3:**
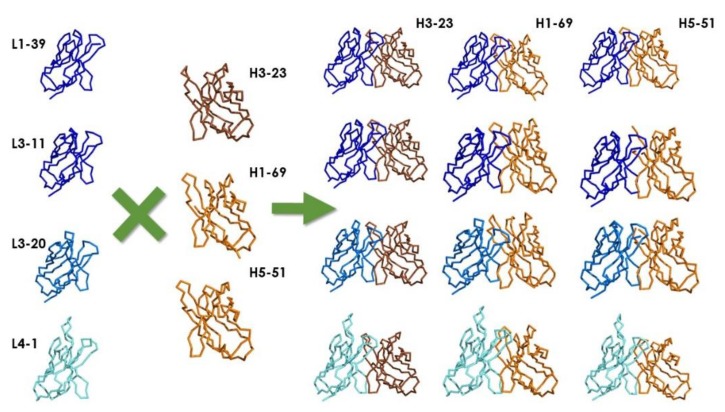
X-ray structures of scaffold combinations used to build Janssen Bio’s pIX Fab libraries. The figure was generated with The Molecular Operating Environment (MOE) from The Chemical Computer Group, Inc. (CCG; www.chemcomp.com) using the structures reported by Teplyakov et al. [77] and deposited at The Protein Data Bank (PDB; rcsb.org) [78]. The colors represent the canonical structures of the scaffolds: Class L2-1 (L1–L3): dark blue; Class L6-1 (L1–L3): blue; Class L3-1 (L1–L3): cyan; Class H1-3 (H1–H2): dark brown; Class L1-2 (H1–H2): light brown. Notice the difference in topography of the antigen-binding site of L1-39, L3-11, and L3-20 (with a short L1 loop) with respect to L4-1(with a long L1 loop).

Janssen Bio’s libraries [29] were the first combinatorial synthetic Fab libraries displayed on pIX instead of the fusion partner pIII that had been used in all other libraries. The libraries were designed with three V_H_ and four V_L_ scaffolds encoded by human germlines (Table 3 and Figure 3). These scaffolds were chosen based on their high usage in the antibody human repertoire and naïve phage display libraries (see Section 4.1. Naïve libraries), as well as on structural considerations. Specifically, the most used canonical structures with a propensity to bind proteins and peptides were represented in the libraries [69,70,71,72,73]. The CDRs were diversified in positions frequently found in contact with protein and peptide targets [37,40]. The diversification regime mirrored the variability of amino acids and frequency observed in the human germline genes and antibodies isolated from natural sources [37,74]. Two sets of libraries were built, one called pIX V2.0 with diversity focused on V_H_ by keeping V_L_ in the germline gene configuration. The other, discussed in the review, was called pIX V3.0 and had diversity in both V_H_ and V_L_ domains.

Recently, Teplyakov et al. [75] determined the structure of the V_H_:V_L_ combinations used in pIX V3.0, which also happened to be common to other synthetic libraries and ALTHEA Gold Libraries™ (Figure 3). Two of the V_L_ scaffolds **(**IGKV1-39 and IGKV3-11**)** have the shortest loop at the LCDR1 observed in human antibodies [76]. Another (IGKV3-20) has an insertion in LCDR1, although it is still a relatively short. The fourth scaffold (IGKV4-01) is the longest LCDR1 seen in the human germline gene repertoire, with an insertion of six residues with respect to IGKV1-39 and IGKV3-11. Altering the length of the LCDR1 from a short to a long loop changes the preference to bind protein or peptide targets, respectively [37,69]. Therefore, the inclusion of these IGKV genes with distinct LCDR1 lengths provided the pIX V3.0 libraries with the potential to recognize diverse types of targets. In V_H_, while the HCDR1 has the same length in all the three scaffolds, and the conformations of the HCDR1 in IGHV3-23 and IGHV5-51 were found to be remarkably similar [75], IGHV1-69 showed large structural variability. This is probably due to two glycine residues in the HCDR1 of IGHV1-69, which provides more conformational freedom. Moreover, HCDR2 has two alternative conformations, one in IGHV3-23*01 and IGHV5-51*01, and another in IGHV1-69. Taken together, the structural variability seen in the pIX V3.0 provided the libraries with distinct topographies and structural diversity to recognize diverse targets.

The thermal stability (Tm) of pIX V3.0 V_H_:V_L_ scaffold combinations in Fab format was also assessed by Teplyakov et al. [75]. All the V_H_:V_L_ scaffold combinations except IGHV3-23*01:IGKV*01 had Tm values above 68 °C. Considering that the least stable domain of the human IgG1 (hIgG1) is the C_H_2 with 68 °C [79], the expectation was that Fabs derived from pIX 3.0 should be developable when converted to hIgG1, which is the therapeutic format most frequently used [12].

PHILODiamond [32] was built with only three scaffolds: one V_H_ (IGHV3-23) and two V_L_s (Figure 3). The V_L_ scaffolds were either kappa (IGKV3-20) or lambda (IGLV3-19*01). PHILODiamond was a new version of the ETH2Gold library [52]. The improvements with respect to the ETH2Gold library consisted of a new HCDR3 design with lengths of four to seven residues diversified with the 20 amino acids per position. In the ETH2Gold library, the HCDR3 had four and six randomized consecutive amino acids. PHILODiamond also has the LCDR3 fully diversified (20 amino acids) in five or six positions. Furthermore, residue 52 of V_H_ was designed to be an asparagine (N) in order to facilitate hydrogen bonding interactions.

### 4.3. Semisynthetic Libraries

As discussed in the previous section, several iterations to improve the quality of synthetic libraries have been performed, with the design of HCDR3 being a constant theme and perhaps the main opportunity for improvement. The HCDR3 is a key element in defining the specificity and affinity of antibodies, but it is also by far the most diverse region of the antigen-binding site and thus difficult to design. The 3D modeling methods [80,81] can predict the structure of all of the CDRs other than the HCDR3 with an accuracy of <1.0 Å [81]. However, no method is currently available to reliably predict the HCDR3 structure, thus limiting our ability to properly design the diversity of this antigen binding-site region.

To avoid any assumption regarding the structure and diversity of the HCDR3, ALTHEA Gold Libraries™ [33] were built with HCDR3 and Joining fragments (H3J fragments) isolated from natural sources. The natural H3J fragments were combined with synthetic scaffolds, which were designed based on human germline genes found to be dominant in the repertoire of human antibodies and numerous scFv and Fab libraries (Table 3). One universal V_H_ scaffold was paired with two V_L_ scaffolds. As in pIX V3.0, we used one V_L_ scaffold to enable the recognition of proteins (IGKV3-20) and other binding peptides (IGKV4-01). Therefore, by using the proper V_L_ scaffold, we hypothesized that antibodies against protein or peptide targets can be selected [37,69]. When used in combination, it would potentially generate antibodies that bind diverse epitopes on a given target. Also, the HFR3 of the universal V_H_ scaffold, being encoded by the IGHV3-23*01 germline gene, naturally binds Protein A of the bacterium *Staphylococcus aureus* [82,83]. The Protein A binding site in the V_H_ domain is formed by discontinuous amino acid stretches distant in the primary sequence brought together by folding. Therefore, Protein A offered a means to select for well-folded scFvs in the construction process of the ALTHEA Gold Libraries™.

**Figure 4:**
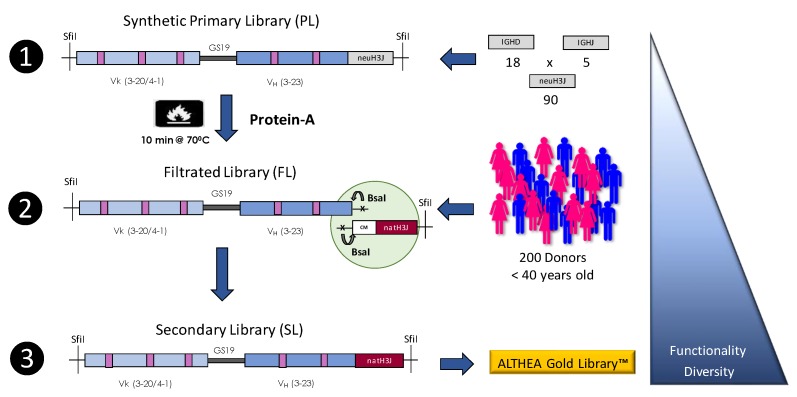
Taken from Valadon et al. [33] with permission. The figure describes the three-step construction process of ALTHEA Gold libraries™. Step 1: Primary synthetic scFv libraries (PLs) based on the two scaffolds 3-20/3-23 and 4-01/3-23, combined with a set of 90 neutral H3J fragments, were assembled and displayed on the phage. Step 2: PLs were heated at 70 °C for 10 min and the surviving stable and well-folded scFvs were rescued by Protein A to create the filtrated libraries (FL). Finally, in Step 3, the diversified scaffolds from the FLs were amplified using PCR with 5′, a universal primer matching CM in the FR-3 of V_H_, and seamlessly assembled with a set of natural H3J fragments that were amplified using RT-PCR from a large pool of 200 donors using the universal primer to build the secondary ALTHEA Gold libraries™ (SLs). The triangle on the right represents how the diversity and functionality of the libraries increased as the three-step process proceeded.

We used a three-step strategy (Figure 4) to generate the libraries. In the first step, fully synthetic primary antibody libraries (PLs) were designed, cloned, and displayed as scFvs on the phage surface. Second, we performed a selection process in which the PLs were submitted to a heat shock and further selected with Protein A for in-frame and thermostable variants. We called the product of this step filtered libraries (FLs). Third, highly functional and highly diverse secondary antibody libraries (SLs) were generated by combining FLs with natural H3J fragments obtained from a large pool of 200 donors. By using this three-step construction process, the functionality of ALTHEA Gold Libraries™, assessed as Protein A binders randomly selected from the libraries, increased from ≈65% in the PLs to ≈85% in the SLs, implying a 20% improvement. In terms of the number of functional clones in a library of 10^10^ variants, it meant 2 × 10^9^ additional antibody sequences to select from.

## 5. Panning Protocols and Targets

Table 4 shows the number of targets, number of clones studied on average per library, hit rate, and affinity of the antibodies selected from the nine libraries discussed in the previous sections. The selections were performed using either solid phase or biopanning with biotinylated targets. In most of the selection protocols, three rounds of panning were used; PHILODiamond proceeded with two rounds of selection and KNU-Fab and ALTHEA Gold Libraries™ proceeded with four.

The number of targets varied from six for pIX V3.0 [29] to 440 for HAL9/10 [15]. As an example of targets, Xoma conducted the panning of the libraries with peptides and proteins, including gastrin 14-mer, β-galactosidase, and TIE-1-Fc chimera. For Ylanthia, mostly proteins were used, such as recombinant human (rh) ErbB4, rhTNFα, and human IgG1 antibodies, to generate anti-idiotypic antibodies. PHILODiamond has been tested with fibronectin, collagen I, tenascin-C (BCD), and human matrix metalloproteinase 1 (MMP1) and 3 (MMP3). MorphoSys tested HuCAL PLATINUM with human Fc fusion proteins, CD20, and human IgG1 antibodies derived from the HuCAL GOLD library. Kim et al. (KNU-Fab) used adhesion molecule (L1CAM), angiopoietin 2 (Ang2), and activation inducible TNF receptor ligand (AITRL). The authors of the ALTHEA Gold Libraries™ explored the functionality of the library with the academic target HEL, and the therapeutic targets human serum albumin (HSA) and TNFα, in addition to another four undisclosed therapeutic targets.

## 6. Enrichment with Positive Clones and Hit Rate

The screening for positive clones after the last round of selection was performed using direct ELISA with the targets. Bovine serum albumin (BSA) or milk was used as specificity controls in most of the selections. The number of clones assayed for binding varied widely. ALTHEA Gold Libraries™ and KNU-Fab libraries screened 45–90 colonies picked at random from the final round of panning. Xoma assayed several hundred colonies, and thousands were screened for HuCAL PLATINUM, Ylanthia, and HAL9/10. The average percentage of positive clones also varied from approximately 20% to close to 60% in XscFv2. For some targets, for instance collagen I when panning PHILODiamond, the result was only a few (1%) positive clones. Other targets, such as Tie2/Ang1 and Tie2/Ang2, yielded as much as 85% and 88% positive clones, respectively, when panning was performed with the XscFv2 library. A lower percentage of positive and unique clones was observed in HuCAL PLATINUM with challenging targets, e.g., anti-idiotype selection, where the unique epitopes on the target were limited to the antigen-binding site of the antibodies [30].

Most of the authors defined the hit rate as the number of positive and unique sequences. MorphoSys (HuCAL PLATINUM and Ylanthia) counted unique clones as HCDR3 sequences. Despite these differences, the average hit rate was remarkably similar (≈10%) in all of the libraries, ranging from 2% to 40%. An overall similar hit rate regardless of the library implies that, as the number of screened clones increases, the number of unique clones obtained also increases. Standard screenings involving ten 96-well regular ELISA plates (≈1000 clones) are thus expected to generate 100 or more unique antibodies, as shown by HuCAL PLATINUM [30]. A larger number of unique clones also means higher diversity in the selected antibody variants, which in turns translates into an increased number of better functional and more developable antibodies. A side-by-side comparison of HuCAL GOLD and PLATINUM [30] showed no significant differences in performance of the libraries in terms of positive clones. However, a more diverse pool of unique antibodies was identified in the PLATINUM selections when compared to the outcome from GOLD. As mentioned above, PLATINUM is an advanced generation of GOLD, with several improvements such as removal of developability liabilities and a better HCDR3 design.

## 7. Affinity of the Selected Antibodies

Higher affinity antibodies are desirable or even required for many therapies. Higher affinity antibodies may also require lower doses, which has a direct impact on lowering the cost of goods and treatment, among other factors. Therefore, since the first applications of the phage display methodology to therapeutic antibody discovery, affinity has been one of the most relevant success criteria to evaluate the performance of a library [17]. All of the libraries in Table 1, except PHILODiamond and KNU-Fab, reported sub-nM binders. Antibodies obtained from PHILODiamond K_D_ values typically ranged between 9 and 150 nM. Of note, the affinity was measured with monomeric scFv fragments. Thus, it could be expected that some of these binders would have reached affinity closer to or below nM when measured as IgG due to the avidity effect. KNU-Fab, on other the hand, converted three Fabs to IgGs. The affinity of one of them for the human and mouse targets were 7.3 nM and 14 nM, respectively, as determined using competition ELISA. Another antibody had a K_D_ value for the mouse target of 1.7 nM.

## 8. Developability

Nearly a decade ago, the concept of developability was applied to antibody drug development [18]. Developability encompasses the design principles and experimental assessment of the characteristics a molecule should meet to be further developed or manufactured, formulated, and stabilized in order to achieve the desired therapeutic effects. Developability is affected by multiple factors, including the intrinsic biophysical and biochemical properties of the molecule, as well as by extrinsic parameters such as ionic strength, pH, and formulation additives. As more antibodies have reached the market, and many have failed to perform in preclinical development and clinical trials, it has been realized that antibodies selected from diverse libraries, despite having the desired specificity and affinity, often tend to fail during the formulation and manufacturing development processes due to suboptimal biophysical properties [84,85]. This includes glycosylation of the antigen-binding site residues, which can impair binding. This is particularly critical for the antibodies selected for phage display libraries as *E. coli* does not glycosylate proteins. Thus, variants with glycosylation sites in the CDRs could be selected during the phage display discovery campaign but would lose binding when converted and expressed in mammalian cells for further characterization. Unpaired cysteines can lead to scrambled disulfide bridges and thus generating covalent aggregates. Moreover, antibodies undergo posttranslational modifications (PTM) of amino acids such as asparagine (N) deamidation, methionine (M) oxidation, and aspartic acid (D) isomerization [79]. These amino acid chemical modifications can result in heterogeneity in the antibody preparation and/or a lack of potency if said amino acids are involved in the antigen interaction. In other instances, exposure of tryptophan (W) to the solvent can induce aggregation, thus leading to immunogenic reactions or lack of solubility at concentrations required for the therapeutic indication [85].

A closer look at the antibody sequences from naïve libraries indicate that some of the selected antibodies encoded developability liabilities. For example, the only human functional gene of the IGHV7 family (IGHV7-4-1*01) was selected with frequencies of 0.4% and 0.7% in HAL10 and HAL9, respectively. This human gene has an unpaired cysteine in HFR3 (UniProtKB: A0A0J9YVY3), which may lead to scrambled disulfide bridges. Two other genes from the IGKV1 family (IGKV1-8*1 and IGKV1D-8*1) have an N-glycosylation site at LFR3 and were selected in HAL10 with a frequency of 0.8%. Glycosylated antibodies other than those with the typical N-glycosylation at the C_H_2 domain are not taken to further development [79] since they may lead to heterogenicity during the manufacturing process. Therefore, these genes compromised the effective size and functionality of the libraries and/or perhaps competed with developable sequences during the selection process. Obviously, obtaining developable antibodies from naïve repertoires can be circumvented by increasing the number of antibodies studied during the screening process (see below), and/or by scanning the sequences and removing the sequences with unwanted developability liabilities, and/or assessing the developability experimentally [19]. Nevertheless, more screening and/or additional testing entails higher costs and longer development times.

Recent generations of synthetic and semisynthetic libraries have incorporated filters to remove developability liabilities in the design phase. Also, biophysical assays have been applied to assess the developability of the scaffolds used as the foundations of the libraries. For example, in the construction of Ylanthia [31], several developability assays were used to select for the most developable scaffolds from a set of 400 initial V_H_:V_L_ pair combinations. Interestingly, after selection using diverse targets, most of the molecules closely preserved the biophysical features that were characteristic of the parental V_H_:V_L_ scaffold pairs, whereas slight biophysical deviations were attributed to the influence of certain HCDR3 sequences [31]. The construction process of ALTHEA Gold Libraries™ included a developability filter, as it followed a filtration process consisting of a thermal shock at 70 °C. This process led to a ≈20% increase in thermal stability of 70 °C or above of the antibodies selected using diverse targets [33].

## 9. Current Opportunities and Challenges

The phage display methodology has been shown to be a valuable, robust, and efficient platform to discover and develop human therapeutic antibodies. Until recently, however, the commercial use of phage display was limited to a few companies holding their technology patents [16]. In fact, to the best of our knowledge, the libraries listed in Table 1 are not available for research purposes. Nevertheless, most of the patents covering phage display have expired in Europe and the United States [16], offering a great opportunity to freely use phage display. As this methodology is becoming a commodity, several companies are licensing phage display antibody libraries such as ALTHEA Gold Libraries™ (https://www.globalbioinc.com/Services/). Also, Bio-Rad Laboratories is offering discovery services based on HuCAL PLATINUM (https://www.bio-rad-antibodies.com/hucal-ordering-information.html) at a relatively low cost. On the other hand, a number of phage display antibody libraries generated before 2010 (not reviewed here but widely validated) are available to academic laboratories at The University of Cambridge or the Scottish Biologics Facility (https://www.abdn.ac.uk/sbf/about/sbf-libraries/). These libraries include the synthetic antibody libraries known as Tomlinson’s libraries [86], built at the UK Medical Research Council and a human naïve library of over 10^10^ human antibodies generated by John McCafferty and his colleagues. Tomlinson’s synthetic libraries have produced specific and high affinity antibodies for a number of targets [87,88], whereas McCafferty’s naïve library has been used to select, screen, and sequence around 38,000 recombinant antibodies against almost 300 antigens.

Regarding opportunities for improving current libraries, an ideal antibody library should generate diverse, highly specific, and high affinity antibodies, as well as developable molecules with a minimum of effort, i.e., only two to three rounds of panning. An important parameter and perhaps the simplest one to be considered when building an antibody phage display library is its size, as it correlates with the probability of selecting higher affinity antibodies, as predicted by Perelson [34]. The libraries described in Table 1 reached the maximum possible size of ≈10^11^ antibody variants. With these library sizes, diverse, highly specific, and sub-nM binders have been obtained from nearly all of the libraries, regardless of the antibody repertoire type, be it naïve, synthetic, or semisynthetic. The effective size of the libraries was on average 85%, meaning that there is room for a 15% improvement, assuming a 100% functionality could be reached. A 15% improvement in a repertoire of 10^11^ molecules implies 1.5 × 10^10^ additional antibody variants to select from.

To improve the effective size of the libraries, better DNA synthesis methods can be applied to increase the percentage of ORFs by reducing stop codons, frameshifts, and redundant and/or unwanted amino acids in the targeted positions for diversification. Current synthesis platforms, such as Twist Bioscience’s silicon-based DNA synthesis platform, enables the precise synthesis of variants with a uniform incorporation of variants in each library, as validated by next-generation sequencing (https://www.twistbioscience.com/libraries_poster_precisionsynthesis). This precise synthesis method can be combined with libraries of antibody molecules with an enhanced developability profile. This can be done by incorporating better developability prediction methods in the design phase of the libraries. In this regard, new in silico tools [89] can identify antibody sequences that have anomalous developability values compared with therapeutics antibodies. This metric, called therapeutic antibody profiling (TAP), builds a downloadable structural model of an antibody V sequence and tests it against guideline thresholds of five calculated measures likely to be linked to poor developability. Therefore, by generating each possible variant of an antibody library in silico and filtering out those with poor TAP, non-developable variants can be replaced with developable ones.

Finally, it is worth mentioning that formats other than scFv or Fab, such as single domain antibodies (sdAbs) or nanobodies, have been widely tested and successfully used to build phage display libraries for the discovery and optimization of antibody-based drugs [90,91,92]. These formats have been inspired by V_H_H Camelidae antibodies [93] and shark’s new antigen receptors (NARs) [94]. Due to its size and high stability, nanobodies have found several therapeutic applications, and therapeutic molecules, such as caplacizumab from Ablynx [95], have recently been commercialized. On the other hand, display platforms other than phage have been developed and are used to discover therapeutic antibodies. These platforms include ribosome [96], bacteria [97], and mammalian [98] display methods. Each of these platforms has advantages and disadvantages with respect to phage display, with yeast being one of the most broadly used display platforms [99]. Yeast display has proven to be an efficient means to isolate antibodies with very high affinity, e.g., in the low femto-molar range [100]. Moreover, while phage is limited to the display of antibody fragments, e.g., sdAb, scFv, or Fab, yeast enables the display of full IgG antibodies with glycosylation. Since the end therapeutic product is commonly an IgG and its efficacy and toxicity are an interplay between target epitope, affinity, Fc isotype, and glycosylation, yeast display has become a suitable platform for efficient therapeutic discovery and development [101]. In fact Adimab, an antibody therapeutic discovery company based on yeast display technology, recently announced the biologics license application (BLA) approval in China for an antibody against PD-1 to treat Hodgkin’s lymphoma (https://www.fda.gov/vaccines-blood-biologics/development-approval-process-cber/biologics-license-applications-bla-process-cber).

## 10. Concluding Remarks

Since the seminal works by George Smith [1] and McCafferty et al. [6] over three decades ago, the field of antibody engineering has seen remarkable progress in the discovery and development of therapeutic antibodies. This progress has been built and is in part the product of several generations of phage display antibody libraries. In the previous sections, we reviewed nine of the libraries generated over the last decade. The goal was to assess the state of the art in phage display antibody discovery and outline commonalities and differences among the diverse libraries. The libraries reached 10^10^–10^11^ antibody variants, the maximum possible size. The effective size, understood as the percentage of display, was 50–90%, with most of the libraries reaching 85%. Such libraries yielded nM or sub-nM binders. Interestingly, the average hit rate of the libraries was similar (≈10%), regardless of the nature of the antibody repertoire—be it naïve, synthetic, or semisynthetic—used as the substrate to build the libraries. The main difference seemed to be in the diversity and developability profiles of the antibodies. While naïve libraries have the advantage of capturing the natural repertoire of antibodies, some antibodies selected from these libraries may bear developability liabilities. To circumvent this limitation, synthetic and semisynthetic libraries have undergone several iterations to improve their design, particularly at the HCDR3. These new libraries, together with the recent expiration of patents in the field, should allow academic laboratories and small biotech organizations the free use of phage display methodology. The widespread use of antibody phage display should foster innovation, further the exploration of diverse and novel targets, and generate novel incremental improvements in the phage display methodology.

## Figures and Tables

**Table 1:** Phage display antibody libraries discussed in this review.

Library Name	Company/Laboratory	Repertoire	Display Format	Size (a)	Reference
XFab1	Xoma	Naïve	Fab	3.1 × 10^11^	[26]
XscFv2	Xoma	Naïve	scFv	3.6 × 10^11^	[26]
HAL9/10	TU-IB (b)	Naïve	scFv	1.5 × 10^10^	[27]
KNU-Fab	KNU (c)	Naïve	Fab	3.0 × 10^10^	[28]
pIX V3.0	Janssen Bio	Synthetic	Fab	3.0 × 10^10^	[29]
HuCAL PLATINUM	MorphoSys	Synthetic	Fab	4.5 × 10^10^	[30]
Ylanthia	MorphoSys	Synthetic	Fab	1.3 × 10^11^	[31]
PHILODiamond	ETH Zurich	Synthetic	scFv	4.1 × 10^10^	[32]
ALTHEA Gold Libraries	GlobalBio/ADL	Semisynthetic	scFv	2.1 × 10^10^	[33]

(a) Library size is expressed as number of colony forming unit (cfu) after the transformation of *E. coli* with library DNA; (b) TU-IB: Technische Universität Braunschweig, Institut für Biochemie, Biotechnologie und Bioinformatik; (c) KNU: Kangwon National University.

**Table 2:** Quality of the phage display antibody libraries.

Library Name	ORF (%)		Display (%)	
	Kappa	Lambda	Kappa	Lambda
XFab1	76	85	70	85
XscFv2	74	66	71	58
HAL9/10	-	-	-	-
KNU-Fab	91	N/A	-	N/A
pIX V3.0	46 (28–72)	N/A	77 (51–90)	N/A
HuCAL PLATINUM	85–97	75–93	-	-
Ylanthia	82	82	-	-
PHILODiamond	93	93	90	90
ALTHEA Gold Libraries™	85	N/A	83–85	N/A

N/A: non-applicable since these are kappa only libraries; “-” means the information is not reported. In parenthesis range of ORFs or Display percentage reported for the Libraries.

**Table 3:** V_H_, Vκ, and V_λ_ scaffolds used as foundations for the synthetic and semisynthetic libraries. We did not include HuCAL PLATINUM in the table since the scaffolds are not natural germline genes but consensus sequences.

Ylanthia			pIX V3.0			PHILODiamond			ALTHEA Gold Libraries		
VH	Kappa	Lambda	VH	Kappa	Lambda	VH	Kappa	Lambda	VH	Kappa	Lambda
1–18	1–6	1–40	1–69	1–39	N/A	3–23	3–20	3–19	3–23	3–20	N/A
1–46	1–9	1–47	3–23	3–11						4–1	
1–69	1–12	1–51	5–51	3–20							
3–7	1–15	2–11		4–1							
3–11	1–27	2–23									
3–15	1–39	3–1									
3–21	3–15										
3–23	3–20										
3–53											
3–74											
5–51											
6–1											
HCDR3 (loop lengths)											
4–25			3–15			4–7			Natural (1–25)		

Color code: Orange: V_H_ scaffolds paired with both Vκ, and V_λ_. Yellow: IGHV3-23 is paired with both Vκ, and V_λ_ and used in all the libraries. Dark green: scaffolds used in all the libraries. Light green: scaffolds used in more than one library. N/A: non-applicable; these are kappa only libraries.

**Table 4:** Panning and performance of the phage display antibody libraries.

Library	Panning			Screening			K_D_ (nM)
	Number of Targets	Selection Method (a)	Rounds	Number of Assayed Clones	Positive Clones (%)	Hit Rate (%)	
XFab1	7	PS, SP	3	757 (465–930)	28 (10–16)	7 (3–11)	0.90 (0.02–2.10)
XscFv2	7	PS, SP	3	797 (558–930)	58 (16–88)	10 (5–17)	0.46 (0.01–1.50)
HAL9/10	440 (b)	-	-	20,000	-	17 (c)	-
KNU-Fab	10	PS, SP	3–4	94	-	5 (2–9)	37.80 (1.70–130.00)
pIX V2/3	6	SP	4	94	-	11 (5–18)	1.00 (0.20–20.00)
HuCAL PLATINUM	6	PS, SP	3	10,000	-	15 (10–30)	0.24 (0.002–10.00)
Ylanthia	9	PS, SP	3	4050 (2900–5200)	1–49 (range)	1–37 (range)	0.70–190 (Fab)
PHILODiamond	15	PS, SP	2–3	94	22 (1–61)	-	9.00–150.00 (scFv)
ALTHEA Gold Libraries™	7	PS, SP	3–4	43–90	20 (10–46)	10 (2–40)	1.00 (0.09–20.00)

(a) SP: Solid phase. PS: Panning in solution; (b) Taken from Frenzel et al. [16]; (c) Calculated based on Table 2 of Frenzel et al. [16]. These authors report 2637 unique antibodies isolated from 440 unique targets (440/2637 = 17%).
